# Associations of plasma 8-iso-prostaglandin F_2α_levels with fasting blood glucose (FBG) and intra-abdominal fat (IAF) area in various Glycometabolism populations

**DOI:** 10.1186/s12902-021-00879-3

**Published:** 2021-10-28

**Authors:** Ning Ma, Yujian Zhang, Binbin Liu, Xiaojiao Jia, Rui Wang, Qiang Lu

**Affiliations:** 1 Department of Endocrinology, First Hospital of Qinhuangdao, No 258, Wenhua Road, Haigang District, Qinhuangdao, 066000 Hebei Province China; 2 Department of Orthopedics, First Hospital of Qinhuangdao, Qinhuangdao, 066000 Hebei Province China; 3 Department of Functional examination department, First Hospital of Qinhuangdao, Qinhuangdao, 066000 Hebei Province China

**Keywords:** Oxidative-stress, 8-iso-prostaglandin F_2α_, Abdominal fat area, Glycometabolism, Diabetes mellitus

## Abstract

**Background:**

This study aimed to investigate the differences in oxidative stress (OS) levels represented by 8-iso-prostaglandin F_2α_ (8-iso-PGF_2α_) and analyze its correlation with the intra-abdominal fat (IAF) area and the glycolipid index.

**Methods:**

We recruited a total of 160 eligible subjects. According to the blood glucose levels and the T2DM duration, subjects were divided into three groups: Type 2 Diabetes (T2DM) group, Prediabetic group, and Normal glucose-tolerance (NC) group, containing 66, 41, 53 patients, respectively. T2DM groups were additionally divided into a new-onset T2DM group including 29 patients and a non-new-onset T2DM group including 37 patients. General clinical data and biochemical indicators were collected. Intra-abdominal fat (IAF) was measured by MRI. 8-iso-PGF_2α_ was measured by ELISA.

**Results:**

Compared with the NC group, levels of systolic blood pressure (SBP), waist-to-hip ratio (WHR), FBG, 2 h postprandial glycemia(2hPG), 2 h insulin (2 h INS), IAF area, HOMA-IR, and 8-iso-PGF_2α_ increased, and high-density lipoprotein cholesterol (HDL-C) decreased in T2DM groups and Prediabetic group (*P* < 0.05). The 2 h INS level was the highest in the Prediabetic group; 2hPG, and IAF area were the highest in the new-onset T2DM group; WHR, FBG, HOMA-IR and 8-iso-PGF_2α_ were the highest in the non-new-onset T2DM group. Multiple stepwise regression analysis identified IAF area and FBG as the strongest and independent determinant of 8-iso-PGF_2α_ (*P* < 0.01).

**Conclusions:**

In various glycometabolism populations, 8-iso-PGF2α is significantly correlated with FBG and IAF, this suggests that high blood glucose and abdominal obesity can increase the damage related to the OS in vivo.

## Background

Diabetes is one of the major health problems worldwide, with an incidence that has been increasing at an alarming rate. The number of people suffering from diabetes has increased approximately four times from 1980 to 2014, affecting 5% of the world’s population [1]. Diabetes is usually associated with cardiovascular and hyperglycemia-specific microvascular complications.

Type 2 diabetes is the most common type of diabetes that mainly affects adults. Chronic inflammation and oxidative stress (OS) have been suggested as important factors in the occurrence and development of Type-2 Diabetes (T2DM) [2, 3]. Oxidative stress is defined as the imbalance between stronger oxidation and weaker antioxidation [4], resulting in neutrophils’ inflammatory infiltration, protease’s secretion together with a large number of oxidation intermediates. Oxidative stress increases the mitochondrial electron transport chain (ETC) activity and reactive oxygen species (ROS) production [5, 6]. At low doses, ROS are considered to be essential for the regulation of normal physiological functions involved in development such as cell cycle and proliferation, differentiation, migration and cell death. But surplus ROS cause oxidative damage to proteins, nucleic acids, lipids, membranes and organelles, which can lead to activation of cell apoptosis [7]. Similarly, glucose toxicity induces higher levels of OS by increasing ROS levels and decreasing antioxidant activity [8, 9]. OS can accelerate the progression of type 2 diabetes, and it is of great significance to judge the severity of type 2 diabetes. The prostaglandin, 8-iso-prostaglandin F_2α_(8-iso-PGF_2α_), is an isoprostane produced by the non-enzymatic peroxidation of arachidonic acid in membrane phospholipids that can also be used as an indicator of enhanced rate of lipid peroxidation [10, 11]. Previous studies have demonstrated that 8-iso-PGF_2α_ can be used as a relevant factor for predicting the capacity of blood glucose control and internal oxidation in patients with T2DM, as well as a reliable laboratory index for predicting prediabetes (impaired glucose tolerance) [12–14]. Overall, 8-iso-PGF_2α_ is a valuable biomarker for the evaluation of the level of peroxidation in various glycometabolism populations.

This study aimed to investigate the differences in oxidative-stress levels represented by 8-iso-PGF_2α_ and analyze its correlation with intra-abdominal fat (IAF) area (obtain by MRI scan) and the glycolipid index.

## Methods

### General data

A total of 160 patients who underwent a physical examination at the Endocrine Department of First Hospital of Qinhuangdao between Nov. 2017 and Nov. 2018 were enrolled in this study. The classification of glucose metabolism status was based on the diagnostic criteria for diabetes published by the World Health Organization in 1999, ① normal blood glucose: fasting blood glucose < 6.1 mmol/L and blood glucose < 7.8 mmol/L 2 h after meals ② impaired fasting blood glucose alone: 6.1 mmol/L ≤ fasting blood glucose < 7.0 mmol/L and 2 h blood glucose < 7.8 mmol/L ③ impaired glucose tolerance alone: fasting blood glucose < 6.1 mmol/L and 7.8 mmol/L ≤ 2 h blood glucose < 11.1 mmol/L ④ impaired fasting blood glucose and impaired glucose tolerance at the same time: 6.1 mmol/L ≤ fasting blood glucose < 7.0 mmol/L and 7.8 mmol/L ≤ 2 h blood glucose < 11.1 mmol/L ⑤ diabetes: fasting blood glucose ≥7.0 mmol/L or 2 h blood glucose ≥11.1 mmol/L. Among them, impaired fasting glucose and/or impaired glucose tolerance are collectively referred to as impaired glucose regulation, also known as prediabetes.

All subjects received an oral glucose tolerance test (OGTT) and were consequently divided into new-onset T2DM group (FBS ≥ 7.0 mmol/L or 2hPG ≥ 11.1 mmol/L), non-new-onsetT2DM group (FBS ≥ 7.0 mmol/L or 2hPG ≥ 11.1 mmol/L), Prediabetic group (6.1 mmol/L ≤ FBS<7.0 mmol/L or 7.8 mmol/L ≤ 2hPG<11.1 mmol/L) and normal glucose-tolerance (NC) group (FBS<6.1 mmol/Land2hPG<7.8 mmol/L), including 29, 37, 41, 53 patients, respectively. Patients in the non-new-onset group were those whose disease duration spanned from 5 to 10 years. Acute glucose metabolism disorder, acute/chronic infection, liver/kidney diseases were excluded in all subjects. All subjects did not use probucol, VE, VC, folic acid, coenzyme Q10 and other anti-oxidative stress drugs. For the non-new-onset group, insulin secretagogues and thiazolidinediones (TZDs) were stopped 24 h before haemospasia to remove the effects on βcell function with limosis and sugar-loaded.

The ethics committee of our hospital approved this study (IRB number 2018H010), and all subjects signed the informed consent.

### Methods

Age, gender, height, weight, blood pressure, waist circumference, hip circumference, and other clinical data were collected from all subjects. Body mass index (BMI) was calculated as follows: *BMI = weight/height*^*2*^; the waist-to-hip ratio (WHR) was calculated using the following formula: *WHR = WC/HC*.

Each patient underwent an OGTT with 75 g of oralanhydrous glucose that was initiated at 8:00 am, and bloodsamples were taken at the same time and after 2 h. Plasma glucose levels were measured using the glucose oxidase method (Yellow Springs Instruments, Yellow Springs, OH), serum lipid levels were measured using an autoanalyzer (model 747–200; Roche-Hitachi), and insulin level was measured using chemiluminescence (Roche 2010 Elecsys). The following equations were used for the HOMA–IR index: [fasting insulin level (mIU/L) × fasting glucoselevel (mmol/L)]/22.5. IR was represented byHOMA–IR [15].

Intra-abdominal fat (IAF) was measured at an L4/5 level using MRI. Briefly, all subjects were placed in a supine position. Then, subjects were instructed to hold their breath while their umbilical plane was scanned.

8-iso-PGF_2α_ was measured by enzyme-linked immunosorbent assay (ELISA, kit produced in BIM, USA, Cat. No.B160040; average intra-assay discrepancy< 15%, inter-assay discrepancy< 10%) following the manufacturer’s instructions.

### Statistical analysis

SPSS13.0 was used for statistical analysis; values were expressed as mean ± standard deviation. Analysis of variance (ANOVA) was used for comparison among multi-groups; the SNK test was used for comparison among intra-groups. Pearson correlation was used for correlation analysis; multiple linear regression analysis was used for screening risk factors. Logistic regression analysis was used to predict the risk factors, and ORs were calculated using the tertile method. *P* < 0.05 was considered statistically significant.

## Results

### Comparison of clinical data and biochemical indicators among different groups

There was no difference in age, BMI, DBP, WC, HC, TG, TC, LDL-c and FINS among groups (*P* > 0.05); while SBP, WHR, FBG, 2hPG, 2 h INS, IAF area, HOMA-IR, and 8-iso-PGF_2α_ were all increased, and HDL-C decreased in Prediabetic group, New-onset T2DM, and non-new onset T2DM groups compared to the NC group (*P* < 0.05). The 2 h INS level was the highest in the Prediabetic group; SBP, 2hPG, and IAF area were the highest in the new-onset T2DM group; WHR, FBG, HOMA-IR and 8-iso-PGF_2α_ were the highest in the non-new-onset T2DM group (Table 1).

**Table 1:** Comparison of clinical data and biochemical indicators among groups

Variables	NC group	Prediabetes group	New-onset T2DM group	Non-new-onset T2DM group	F	*P*
N(M/F)	53 (27/24)	41 (22/19)	29 (16/13)	37 (18/19)	–	–
Age (year)	55.42 ± 9.71	57.59 ± 7.71	57.86 ± 9.27	58.67 ± 6.46	1.173	0.322
BMI (kg/m^2^)	25.53 ± 3.23	27.00 ± 2.73	25.73 ± 2.94	26.29 ± 3.31	1.691	0.171
SBP (mmHg)	119.42 ± 14.86	128.15 ± 15.77#	131.62 ± 15.90#	128.30 ± 14.74#	4.853	0.003
DBP (mmHg)	77.63 ± 10.83	81.06 ± 8.99	82.72 ± 12.41	78.43 ± 10.28	1.753	0.159
WC (cm)	89.25 ± 10.58	90.76 ± 8.52	89.78 ± 9.79	93.53 ± 7.72	1.614	0.189
HC (cm)	101.29 ± 8.31	102.71 ± 6.38	99.91 ± 6.44	99.45 ± 6.92	1.420	0.239
WHR	0.88 ± 0.06	0.88 ± 0.06	0.90 ± 0.05	0.94 ± 0.06#&*	9.518	0.000
TG (mmol/L)	1.77 ± 1.04	1.79 ± 0.98	1.76 ± 0.86	2.13 ± 1.91	0.720	0.542
TC (mmol/L)	5.29 ± 1.17	5.05 ± 0.94	5.41 ± 1.18	5.55 ± 1.57	1.028	0.382
HDL-C (mmol/L)	1.47 ± 0.36	1.37 ± 0.35	1.45 ± 0.32	1.26 ± 0.24#*	3.193	0.025
LDL-C (mmol/L)	2.78 ± 0.91	2.68 ± 0.71	2.75 ± 0.78	3.04 ± 1.07	1.158	0.328
FBG (mmol/L)	5.03 ± 0.49	5.49 ± 0.71	6.06 ± 1.19#	8.38 ± 2.85#&*	34.870	0.000
2hPG(mmol/L)	6.20 ± 1.11	9.24 ± 1.13#	13.76 ± 3.03#&	13.35 ± 5.22#&	57.991	0.000
FINS (mIU/L)	8.01 ± 4.42	8.91 ± 4.56	7.60 ± 3.11	8.76 ± 6.34	0.565	0.639
2 h INS (mIU/L)	32.13 ± 20.90	67.00 ± 57.71#	47.93 ± 23.94#&	31.45 ± 34.29&*	7.634	0.000
IAF area (cm^2^)	83.27 ± 41.55	99.50 ± 27.95	109.07 ± 43.93#	104.25 ± 33.94#	3.697	0.013
HOMA-IR	1.81 ± 1.04	2.18 ± 1.17	2.06 ± 1.00	3.93 ± 4.12#&*	6.987	0.000
8-iso-PGF_2a_(pg/ml)	32.28 ± 20.09	39.02 ± 21.72	42.22 ± 25.44#	50.06 ± 31.41#	3.688	0.013

*Abbreviations*: *BMI* body mass index, *SBP* systolic blood pressure, *DBP* diastolic blood pressure, *WC* waist circumference, *HC* hip circumference, *WHR* waist-hip ratio, *TG* triglyceride, *TC* total cholesterol, *HDL-C* high-density lipoprotein cholesterol, *LDL-C* low-density lipoprotein cholesterol, *FBG* fasting blood glucose, *2hPG* 2 h postprandial glycemia, *FINS* fasting insulin, *2 h INS* 2 h insulin, *IAF* intra-abdominal fat, *HOMA-IR* homeostasis model assessment-insulin resistance, *8-iso-PGF*_*2a*_ 8-iso-prostaglandin F2a

^#^,compared with NC group, *P* < 0.05; ^&^,compared with prediabetes group, *P* < 0.05;*, compared with new-onset T2DM group, *P* < 0.05

### Correlation between 8-iso-PGF_2α_ (blood) and different indexes in various impaired glucose-regulation populations

Pearson correlation analysis (Table 2) showed that 8-iso-PGF_2α_ level in blood was positively correlated with BMI, WC, HC, WHR, FBG, 2hPG, FINS, IAF area, HOMA-IR (*P* < 0.05), and negatively correlated with HDL-C (*r* = − 0.205, *P* = 0.012).

**Table 2:** Correlation of 8-iso-PGF_2α_ level in blood with visceral-fat area and glucose metabolism, etc.

Variables	r	*P*
Age (year)	0.079	0.340
BMI (kg/m^2^)	0.483	0.000
SBP (mmHg)	0.111	0.178
DBP (mmHg)	0.083	0.316
WC (cm)	0.620	0.000
HC (cm)	0.381	0.000
WHR	0.531	0.000
TG (mmol/L)	0.145	0.079
TC (mmol/L)	0.042	0.613
HDL-c (mmol/L)	−0.205	0.012
LDL-c (mmol/L)	0.115	0.162
FPG (mmol/L)	0.264	0.001
2 h PG (mmol/L)	0.269	0.001
FINS (mIU/L)	0.283	0.001
2 h INS (mIU/L)	0.099	0.242
IAF area (cm^2^)	0.841	0.000
HOMA-IR	0.251	0.002

*Abbreviations*: *BMI* body mass index, *SBP* systolic blood pressure, *DBP* diastolic blood pressure, *WC* waist circumference, *HC* hip circumference, *WHR* waist-hip ratio, *TG* triglyceride, *TC* total cholesterol, *HDL-C* high-density lipoprotein cholesterol, *LDL-C* low-density lipoprotein cholesterol, *FBG* fasting blood glucose, *2hPG* 2 h postprandial glycemia, *FINS* fasting insulin, *2 h INS* 2 h insulin, *IAF* intra-abdominal fat, *HOMA-IR* homeostasis model assessment-insulin resistance

### Regression analysis of influencing factors towards the 8-iso-PGF_2α_ level in the blood

In order to exclude confounding factors and determine which indicators are significantly correlated with 8-ISO-PGF2 α, we selected Age, BMI, SBP, DBP, FBG, 2hPG, FINS, 2H INS, TG, TC, HDL-c, LDL-c, HOMA-IR, WC, HC, WHR, and visceral-fat area as independent variables and 8-iso-PGF_2α_ in blood as a dependent variable in multiple stepwise regression analysis. Additionally, the visceral-fat area and FBG were also included in this equation. Ultimately, following regression equation was established: 8-iso-PGF_2α_ = − 20.783 + 0.543 (IAF area) + 1.326 (FBG), *R*^2^ = 0.711, F = 166.360, *P* = 0.000 (Table 3). The equation suggested that 8-iso-PGF_2α_ was positively correlated with IAF area (β = 0.821, SE = 0.031, *P* = 0.000) and FBG (β = 0.102, SE = 0.612, *P* = 0.032). The higher the fasting glucose, the higher the level of 8-iso-PGF_2α_.

**Table 3:** Multiple linear regression analysis of influencing factors towards the 8-iso-PGF_2α_ level in the blood

Variables	Unstandardized coefficients β	SE	Standardized coefficients β	*t*	*P*	95% *CI* for β
IAF area (cm^2^)	0.543	0.031	0.821	17.512	0.000	0.482 ~ 0.604
FBG (mmol/L)	1.326	0.612	0.102	2.168	0.032	0.116 ~ 2.535
Constant	−20.783	4.488	–	−4.631	0.000	−29.659 ~ −11.908

*IAF* intra-abdominal fat, *FBG* fasting blood glucose, *8-iso-PGF*_*2a*_ 8-iso-prostaglandin F2a

### Multivariate logistic analysis of the different tertiles of 8-iso-PGF_2α_in blood with IAF and FBG

To further confirm the above conclusion, multivariate logistic regression analysis was performed by grouping the tertiles of 8-iso-PGF_2α_ levels, with IAF > 100 cm^2^ as abnormal [16] and FBG > 7.0 mmol/L as abnormal. The 8-iso-PGF_2α_ tertile, BMI, and age (graded by 10 years) were used as independent variables, and the presence or absence of abnormal IAF and abnormal FBG were used as dependent variables. The results of multivariate logistic regression analysis showed that 8-iso-PGF_2α_ could enter the regression equations of abnormal IAF and abnormal FBG, respectively, and neither age nor BMI entered each equation. For each grade increase in 8-iso-PGF_2α_, the odds ratio was 1.222 for abnormal IAF and 1.129 for abnormal FBG. (Table 4).

**Table 4:** Multivariate logistic analysis of the different tertiles of 8-iso-PGF2α in blood with IAF and FBG

Variables	β	P	OR	95% CI
IAF area (cm^2^)	0.200	0.000	1.222	1.138 ~ 1.312
FBG (mmol/L)	0.079	0.000	1.129	1.113 ~ 1.145

*IAF* intra-abdominal fat, *FBG* fasting blood glucose, *8-iso-PGF*_*2a*_ 8-iso-prostaglandin F_2a_

The tertiles of 8-iso-PGF_2α_ levels are 1: <48.6 pg/ml, 2: 48.6 ~ 100.2 pg/ml and 3: >100.2 pg/ml

## Discussion

In this study, we investigated the role of 8-iso-PGF_2α_ in the occurrence and development of T2DM. Our data indicated that increased levels of 8-iso-PGF_2α_ induced by DNA damage caused by oxidative stress, were associated with poor blood glucose control and the development of T2DM in the prediabetic stage (in the stage of impaired glucose tolerance). These data are consistent with Răchişan et al [17], who examined the activity of 8-iso-PGF_2α_ in pediatric patients with type 1 diabetes mellitus.

T2DM is a type of progressive disease characterized by impaired islet β cells and insulin resistance. Following oxidative damage of β cells, the esterified 8-iso-PGF_2α_ increases and, in turn, damages the integrity and fluidity of the β cells membrane, resulting in the wreckage of cell structure, function, and even cell death [7]. The 63% decline in the volume of β cells in obese T2DM patients has been associated with increased β-cells’ apoptosis [18]. In this study, we found a significant increase of 2 h insulin plasma-concentration in the prediabetic group. It is possible that during this stage (early stage), the oxidative-stress damage is obscured by the rise of insulin β-cells secretion. Then, during a late-stage, 8-iso-PGF_2α_ increase, damaging islet cells and decreasing the secretion of insulin. Nonetheless, the target cells also are attacked by it, which may weaken the signal transduction after Insulin-Receptor binding, thus accelerating IR to fire oxidative-stress through the production of oxygen free radicals on mitochondrial respiratory chains. What is interesting is that 2 h INS significantly increased in the prediabetic group and new-onset T2DM group, which is consistent with the results of Pfuetzner et al. [19]. The results show that Intact proinsulin response to glucose loading might indeed be a useful indicator for predicting worsening to diabetes in normal subjects or subjects with impaired glucose tolerance. To sum up, oxidative-stress and its metabolite 8-iso-PGF_2α_ cause IR, which then strengthens the occurrence and development of oxidative-stress response.

The correlation between IAF and metabolic dysfunction has been verified in previous studies. The degree of IAF accumulation can predict the subsequent development of T2DM [20–22], thus emphasizing its essential role of visceral-fat content in diabetes. Besides, accessible adipokines can be secreted by adipose tissues whose elevated amounts in the abdomen may advance this secretion, and consequently lead to an enhancive degree of OS [23]. In white adipose tissue (WAT), mitochondrial OS, and the generation of ROS affect the endocrine and metabolic function of fat cells [24]. Multiple linear regression analysis showed that the IAF area and FBS were the independent influencing factors of 8-iso-PGF_2α_, the risk of abnormal IAF was 1.222 times higher than that of normal and the risk of abnormal FBG was 1.129 times higher than that of normal for each increasing level of 8-iso-PGF_2α_ for trichotomization. A decrease of IAF area and blood-glucose level improves the change of OS in the development of T2DM.

Directions may be targeted by reducing the level of 8-iso-PGF_2α_ in prevention and treatment for T2DM patients. Costacou et al [25] have reported that the use of antioxidants could reduce peroxidation degree (8-iso-PGF_2α_level) in diabetic patients, as well as reduce the possibility of developing coronary heart disease events. Among multitudinous treating strategies to lessen oxidativestress by decreasing8-iso-PGF_2α_level, a meta-analysis verified the exact roles of antioxidants and many other treatments; yet, not all methods have shown to be effective, which demands additional evaluations of the integrity of clinical conditions [26]. It is worth mentioning that clinical interventions for early abdominal obesity and insulin resistance should be used to achieve early diagnosis, treatment and prevention of type 2 diabetes and other related diseases.

There were following limitations to our study. It must be pointed out that the present study is a single-center study, and a limited number of participants were included. In addition, the diagnosis of prediabetes and T2DM was not based on Hemoglobin A1c (HbA1c) level. Therefore, based on these limitations, large-sample, multi-center research is required in the future.

## Conclusions

To conclude, there was a considerable discrepancy among 8-iso-PGF_2α_ levels in glycometabolism populations; 8-iso-PGF_2α_ was significantly correlated with the IAF area and FBG, which implies the role of OS in the occurrence and development of diabetes and diabetes-related complications. The correlation of IAF area and FBG with diabetes-related complications needs further exploration.

## Data Availability

The datasets used and/or analysed during the current study are available from the corresponding author on reasonable request.

## Abbreviations

8-iso-PGF_2α_: 8-iso-prostaglandin F_2α_
IAF: Intra-abdominal fat
NC: Normal glucose-tolerance
T2DM: Type 2 diabetes mellitus
IR: Insulin resistance
HOMA-IR: Homeostasis model assessment-insulin resistance
FINS: Fasting insulin
FBG: Fasting blood glucose
MRI: Magnetic Resonance Imaging
ELISA: Enzyme-Linked Immunosorbent Assay
SBP: Systolic blood pressure
WHR: Waist-to-hip ratio
2 h PG: 2 h postprandial glycemia
2 h INS: 2 h insulin
HDL-C: High-density lipoprotein cholesterol
BMI: Body mass index
WC: Waist circumference
HC: Hip circumference
TG: Triglyceride
HbA1c: Hemoglobin A1c
